# Mapping sex and gender in the landscape of spinal cord injury research: a bibliometric analysis and research framework

**DOI:** 10.1038/s41393-025-01089-7

**Published:** 2025-05-29

**Authors:** Stevan Stojic, Serena Affolter, Gertraud Stadler, Stacey A. Missmer, Juergen Pannek, Jivko Stoyanov, Inge Eriks-Hoogland, Janina Lüscher, Marija Glisic

**Affiliations:** 1 Swiss Paraplegic Research, Nottwil, Switzerland; 2 Institute of Social and Preventive Medicine (ISPM), University of Bern, Bern, Switzerland; 3 Institute of Gender in Medicine (GiM), Charité- Universitätsmedizin Berlin, Berlin, Germany; 4 Department of Epidemiology, Harvard T.H. Chan School of Public Health, Harvard University, Boston, MA USA; 5 Swiss Paraplegic Center, Nottwil, Switzerland; 6 Faculty of Health Sciences and Medicine, University Lucerne, Lucerne, Switzerland

**Keywords:** Epidemiology, Outcomes research

## Abstract

**Study design:**

Bibliometric analysis and conceptual framework.

**Objectives:**

To provide a framework for prioritizing sex/gender research in the field of spinal cord injury (SCI), which can help inform and develop future research directions benefiting both women and men affected by SCI.

**Setting:**

Not applicable

**Methods:**

We searched the Web of Science Core Collection to identify relevant articles. Data was analyzed using the Bibliometrix and VoSviewer tools to provide a macroscopic overview of sex/gender research trends in the field of SCI research. A framework was developed based on the results of bibliometric analyses and literature scoping, engaging professionals with backgrounds in gender medicine, translational medicine, psychology, clinical epidemiology, SCI, and endocrinology.

**Results:**

A total of 1031 documents were included in the analyses. We observed a steady increase in sex/gender related research from 2012, with an annual growth rate of 9.64%. Rehabilitation, epidemiology, obesity, depression, and sex hormones were identified as fundamental and critical topics for advancing sex and gender research in the context of SCI. Among a randomly selected articles, a significant proportion of studies interchangeably used the terms sex and gender. Therefore, we discuss the key overarching themes and terminology that are essential for any study exploring the relevance of sex and gender in health research. We developed a three-step research framework for considering and incorporating sex and gender in research, using SCI as a case in point.

**Conclusion:**

The major principles in current paper can benefit everyone interested in studying sex/gender in the context of health in complex and disabling conditions.

## Introduction

Sex refers to the biological characteristics that differentiate males and females, including chromosomal patterns, reproductive anatomy, and hormonal profiles [1, 2]. Biological differences between males and females are present throughout all bodily systems, influencing every facet of human wellbeing, health and disease [3]. The term “sex assigned at birth” denotes the classification of a newborn as male, female, or intersex, typically based on visible anatomical features. In health research, sex assigned at birth is frequently used as a proxy for biological sex due to its accessibility in administrative records [1, 2]. Gender encompasses the socially constructed roles, behaviors, expressions, and identities associated with being a girl, woman, boy, man, or persons of diverse genders. It influences health at multiple levels. At a structural level, the distribution of power and resources within a society impacts access to healthcare. Gender norms shape behaviors, such as risk-taking or health-seeking actions, and influence exposure to health risks and hazards through the gendered division of labor. Moreover, gender identity exists along a spectrum and may evolve over time, reflecting increasing awareness of sexual and gender diversity [1, 2].

Although sex and gender are recognized as crucial determinants in the patterns of major diseases, disabilities, and mortality, their integration into research practices remains inadequate [4]. Remarkably, the foundation upon which contemporary healthcare policies and practices are built predominantly draws from clinical research conducted in male study populations [4]. Women remain underrepresented in clinical trials across many fields, and analyses that differentiate by sex and gender, including those involving gender-diverse participants, are often neglected without adequate explanation [5]. Further, health research has mostly focused on biological differences between men and women, using a biomedically-centered concept of sex [4]. Thus, the differences between men and women have been predominantly discussed in the context of biological factors only rather than by a mix of socio-cultural and biological factors. Similarly, in pre-clinical studies, male animals have traditionally been used in experiments, yet, the findings are often generalized to both sexes. The funding agencies, including the Canadian Institutes of Health Research (CIHR), the US National Institutes of Health (NIH) and the European Commission implemented various policies to promote equal representation of both sexes in preclinical research and methods for measuring gender aspects of health and inclusion of sex and gender in research have been widely proposed [6, 7].

**Fig. 1:**
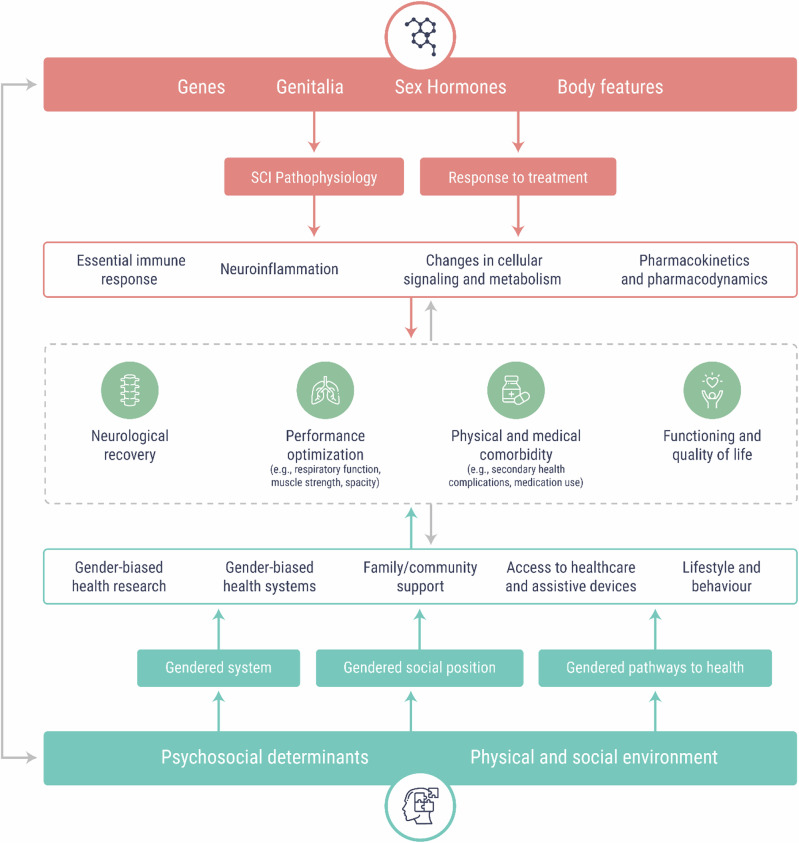
Sex and gender factors influencing recovery and rehabilitation outcomes after SCI. Sex encompasses biological factors determined by sex chromosomes, genetic and epigenetic processes, and sex hormones; whereas gender is a socially constructed concept, encompassing culturally bound norms, roles, and behaviours. For instance, sex can influence initial response to SCI through difference in neuroinflammation or initial immune response. Whereas, use of health care can be affected by a complex interplay between psychosocial determinants and physical and social environment of a person. Sex and gender cannot be observed separately and they constantly influence each other. For instance, hormones can influence behaviour, whereas behaviour and lifestyle can influence epigenetic changes in a sex-specific manner.

Studying sex differences in conditions that predominantly affect men or women is crucial for understanding the biological, hormonal, and psychosocial factors that influence disease prevalence, progression, and outcomes. However, challenges arise when conditions are studied primarily in the predominant sex, leading to gaps in knowledge about the opposite sex. This limits the generalizability of findings and contributes to inequities in diagnosis, treatment, and prevention strategies. For instance, in neuroscience and pharmacology, a bias towards the use of males in pre-clinical studies created a challenge in understanding disorders that affect women more than men, such as major depression and anxiety, potentially leading to the lack of development of effective therapeutics for women [8]. On the other hand, neurotrauma, and in particular, traumatic spinal cord injury (SCI) is an opposite example. In the reproductive period, around 80% of individuals affected by SCI are being male, whereas, pre-clinical studies have been predominantly done on female animal models of SCI [9]. Exclusion of male animals is justified by high mortality rates in male animals following neurotrauma; whereas in clinical research, women are often purposively excluded to make study populations more homogenous and ease interpretation of findings. This mismatch between pre-clinical and clinical studies means that current evidence-based guidelines do not reflect sex-based differences in pathophysiology and treatment responses. Tailoring SCI rehabilitation strategies on such limited demographics and failure to advance our understanding of injury, and treatment in the context of sex and gender-based differences, may lead to suboptimal rehabilitation strategies in both men and women [10]. In Fig. 1 we illustrate a complex interplay between sex- and gender factors influencing neurological recovery, capacity and, risk of medical comorbidity as well as functioning and quality of life in individuals with SCI. Building up on this, we aimed to scope the current scientific literature and describe the research activity concerning the use of sex and gender for more inclusive research in SCI and to provide a framework for research prioritization for sex/gender disaggregated research in the field of SCI, which can help to inform and develop future research directions benefiting both men and women affected by SCI or other complex and disabling health conditions.

## Methods

### Database and search strategy

The study protocol is available at Open Science Framework [11]. We searched the Web of Science (WoS) Core Collection database from inception until 5th October 2023. The search strategy combined terms relevant to SCI and sex and gender, since there were no restrictions concerning study outcomes, no search terms relevant to health outcomes were added to the search string (Appendix I). A search was conducted by Topic, which searches in the title, abstract and keyword fields of WoS records, without language and date restrictions. Publications types including the meeting abstracts, proceeding papers, editorials/letters were excluded from search. The complete set of bibliographic data was downloaded as .ris and .ciw formats.

### Inclusion and exclusion criteria and reference screening

Original studies (observational and interventional), reviews, systematic reviews and meta-analyses including SCI population (human and animal), alone or together with other health conditions, were eligible for inclusion. To filter relevant documents, two screeners reviewed titles and abstracts using the Endnote software. Only studies that clearly articulated the role of sex and/or gender in any context of SCI (e.g. risk factors, pathophysiology of injury, response to treatment) were included. Exclusion criteria included studies done solely in populations other than SCI, conference abstracts, books, proceedings papers and corrected documents. This set was transformed back to WoS format using the Accession Number field via Endnote query and was used for bibliometric and descriptive analyses.

### Data analysis and framework development

The full records of the final dataset were exported to Bibliometrix (Version 3.0 via Biblioshiny) and VOSviewer (version 1.6.18) for bibliometric analyses. Following the title and abstract screening, 50 original articles (excluding meta-analyses) were randomly selected using random number generator in Excel in order to: (1) provide an overview of the type of sex- and gender- disaggregated research strategies; and (2) to explore the prevalence of interchangeable use of sex and gender in SCI research. In the current study we defined a proper sex and gender- disaggregated research as sex-/gender- specific analyses, not merely fitting sex within the multivariable regression models. Sex-specific analyses were considered if male/female or men/women terminology was used (i.e., by simply relying on sex assigned at birth). Gender-specific analyses were considered if gender-based social, economic or lifestyle factors were considered. A research framework was developed based on the results of bibliometric analyses and literature scoping engaging professionals with backgrounds in gender medicine, translational medicine, psychology, clinical epidemiology, SCI and endocrinology.

## Results

**Fig. 2:**
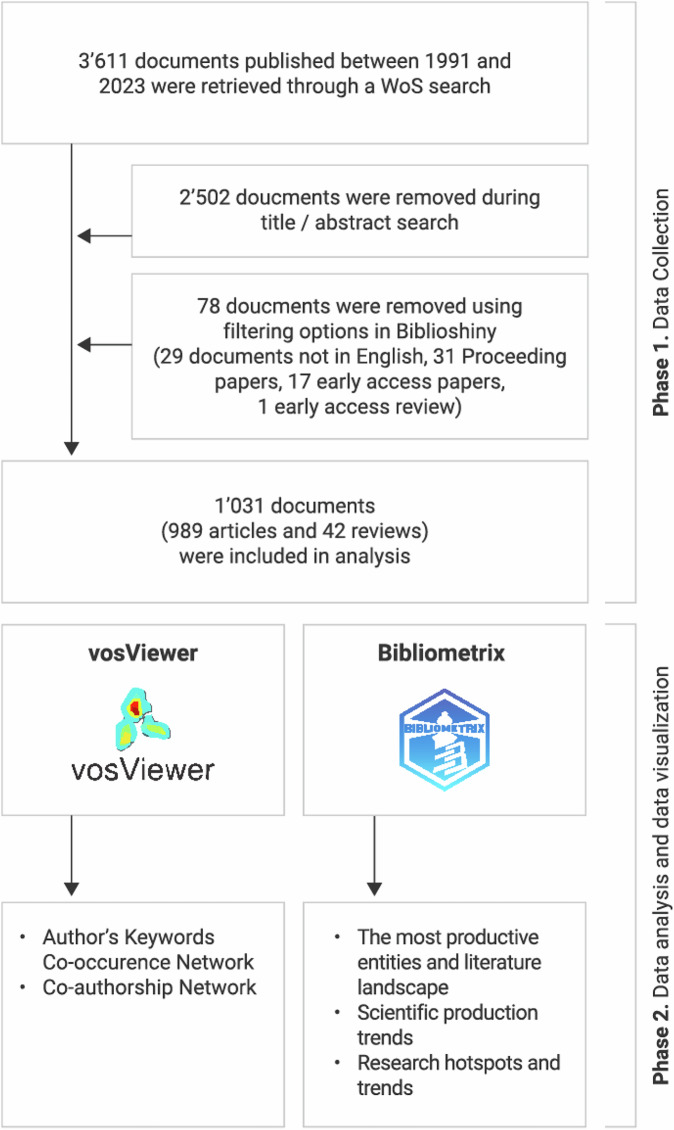
Flowchart of included documents. This figure illustrates the step-by-step process of literature selection and analysis. Phase 1 (Data Collection) shows the identification and screening of documents retrieved from a Web of Science (WoS) search leading to the inclusion of 1,031 documents (989 articles and 42 reviews). Documents were excluded based on title/abstract screening and filtering criteria in Biblioshiny (language, document type, and access status). Phase 2 (Data Analysis and Visualization) displays the tools used: vosViewer for author keyword co-occurrence and co-authorship network analysis, and Bibliometrix for mapping the most productive entities, scientific trends, and research hotspots.

Following title and abstract screening, a total of 1109 documents were exported to Biblioshiny. After additional filtering 78 documents were removed leaving 1031 documents (989 original articles and 42 reviews) to be included in the analyses (Fig. 2). Documents were authored by 5290 scholars, with 18.14% of documents engaging international collaborations (Table 1). Sex and gender research in SCI gained momentum in 2012 and steadily increased—with an annual growth rate of 9.64% (Supplementary Fig. 1). The included documents comprised 1975 authors’ keywords and 2224 keywords plus, which we used in the current analyses. The metadata we used in the analysis was excellent/good quality with the exception of the authors’ keywords quality which was poor (Supplementary Table 1).

**Table 1:** General overview of included data.

Timespan	1991:2023
Sources (Journals, Books, etc)	365
Documents	1 031
Annual Growth Rate %	9.64
Document Average Age	9.34
Average citations per doc	30.4
References	25 111
Document contents	
‐ Keywords Plus	2 224
‐ Author’s Keywords	1 975
Authors	
‐ Authors	5 290
‐ Authors of single-authored docs	24
Authors collaboration	
Single-authored documents	28
Co-Authors per document	6.74
International co-authorships, %	18.14
Document types	
‐ article	989
‐ review	42

### Key sources and the most productive entities

Forty-one percent of the documents (*n* = 1426) were published among the top 10 sources, with Spinal Cord (*n* = 161), Archives of Physical Medicine and Rehabilitation (*n* = 72) and Journal of Spinal Cord Medicine (*n* = 64) being the top three sources (Supplementary Fig. 2a). Based on the analysis of the corresponding author’s country, the USA (*n* = 370, 35.9%), China (*n* = 99,9.6%) and Canada (*n* = 75,7.3%) were the most productive countries (Supplementary Table 2), and the top 3 most productive institutions were the University of Toronto, Harvard University and Tehran University of Medical Sciences (Supplementary Fig. 2b). Supplementary Fig. 2c depicts the top 10 most productive authors. Supplementary Table 3 provides an overview of the ten most cited papers concerning sex/gender research in SCI field with the epidemiology of SCI (e.g., SCI prevalence, incidence, demographic profile of injury etc.) being the most common among the studied topics.

### Popular research teams

**Fig. 3:**
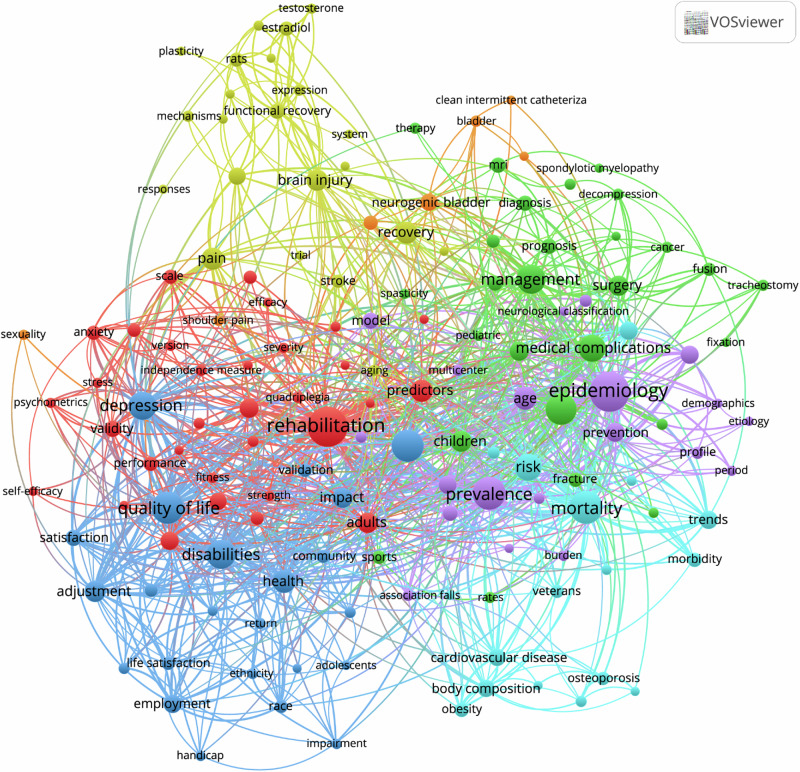
Author’s keywords and key words plus co-occurrence network. Term map showing occurrence of authors keywords and keywords plus in sex/gender SCI research. Phrases occurring in at least 0.2% (8) of the publications were included (*n* = 199). Circle size represents the frequency of occurrence, colour represents the citation per publication, and distance between 2 circles represent how 2 phrases co-occurred in the publications.

Supplementary Fig. 3a provides an overview of the top 50 most frequently used authors’ keywords after removing those keywords related to sex, gender and SCI search terms. “Rehabilitation” (*n* = 103) was the most frequent authors’ keyword followed by “epidemiology” (*n* = 77), “incidence” (*n* = 32), “quality of life” (*n* = 32), and “ mortality “ (*n* = 30). Fig. 3 shows the author’s keywords and keywords plus co-occurrence mapping (co-occurring in >0.2% of publications). The 199 keywords were grouped into eight clusters among which rehabilitation, epidemiology, quality of life and management were the largest. In thematic map (Supplementary Fig. 3b), the themes were grouped into clusters of author’s keywords according to their centrality and density rank values along two axes. Two clusters were identified within the motor themes: one related to international classification of functioning and another related to prediction (including nursing and psychometrics). The lower-right quadrant depicts so-called basic themes, which are fundamental and critical for advancing sex/gender research in SCI including: Rehabilitation, epidemiology, obesity, depression, estrogen and imaging were identified as fundamental and critical for advancing sex/gender research in SCI. Sexuality, risk factors and spinal surgery, were identified as the areas that possibly demand more attention or investment in research. Finally, the upper-left quadrant depicts the niche themes, that are well-developed but not vital for the sex/gender SCI research domain. The osteoporosis, neurogenic bladder and thoracostomy themes were identified as well-developed but not vital for the sex/gender SCI research domain. In our thematic map analysis, we identified “basic themes” based on their centrality and density rank values. We recognize potential limitations associated with this approach. Specifically, the density metric may be disproportionately influenced by the output of a few highly prolific research groups, resulting in artificially elevated density values. Similarly, centrality may be affected by network connections that do not necessarily represent broad thematic relevance but rather reflect strong collaborations or citation networks within specific clusters. These factors could potentially skew the interpretation of basic themes and their significance.

### Evaluation of sex- and gender-disaggregated research

Among the 50 randomly selected articles, the most common topics included epidemiology of SCI (*n* = 16, 32%) comprising incidence, prevalence and sex/gender specific risk factors; followed by exploration of risk factors and management of injury complications (*n* = 14, 28%) including pain, cardiovascular diseases and obesity. One third of the studies interchangeably used sex and gender terms in publications (*n* = 14). Twenty-six studies (52%) aimed to either study sex differences or compare study outcomes across the two sexes and used sex assigned at birth in their analyses. Among the studies which aimed to study the role of gender, all studies with exception of one [12], relayed on sex assigned at birth in their analyses. Sex-disaggregated estimates were available in 38% (*n* = 19) of the studies, whereas the other studies entered sex in regression model as an independent variable.

While this subset of 50 articles provided valuable insights, we acknowledge that this selection may not fully capture the breadth and diversity of SCI research methodologies. Random selection, while reducing selection bias, may lack rigor in ensuring a representative and comprehensive understanding of the field. This limitation is particularly relevant for interpreting trends in sex- and gender-disaggregated research. Future studies could address this by employing systematic or stratified sampling approaches to ensure a broader and more balanced representation of SCI research.

#### Research framework for incorporating sex/gender in SCI research

**Fig. 4:**
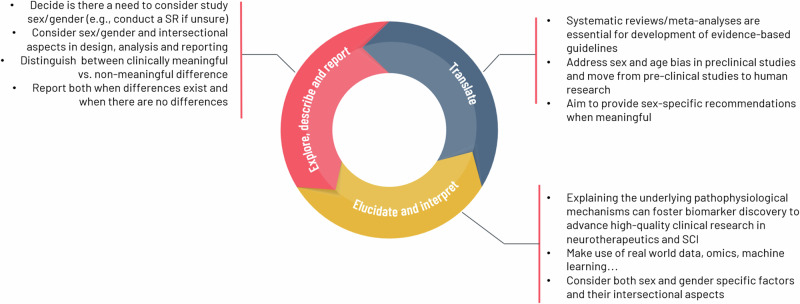
Research framework.

Previous efforts to integrate a sex- and gender-disaggregated approach in health research have led to significant progress across various domains, including health [13–16], environmental health [17], and information systems [18]. Specific guidelines have been developed to address sex and gender considerations in fields such as pediatric pain [19] and rehabilitation [20, 21], aiming to enhance scientific reporting and analysis. In addition, frameworks for evaluating the implementation of sex, gender, and diversity policies have been applied by major national funding agencies globally [6]. A notable contribution is the SAGER (Sex and Gender Equity in Research) guidelines [22], which provide a comprehensive framework for researchers to consistently and transparently report sex and gender in their studies. These guidelines stress the importance of explicitly defining, measuring, and considering sex and gender, ensuring more inclusive and equitable research. By following the SAGER guidelines, researchers can contribute to the accuracy and relevance of sex- and gender-based differences, ultimately advancing the development of personalized, effective health interventions. Building on these efforts, we present a framework that highlights the key aspects of SCI and outlines the major considerations researchers should take into account when addressing sex or gender in their research (Fig. 4).

### Explore, describe and report sex/gender differences

The epidemiology of SCI, response to rehabilitation programs (measured through sensory/motor recovery and functioning), risk of secondary health conditions, provision and utilization of healthcare services, and survival are the key areas to be addressed in future studies. It is important to think of sex (mediated through genetic and hormonal mechanisms), sociocultural aspects of gender and intersectional aspects of sex and gender in study designs, analysis, and reporting of research. Epidemiological research has progressed in sex -sensitivity, recognizing biological differences among sexes, including intersex variations [23]. However, gender-sensitivity remains underdeveloped. This gap involves the consideration of societal norms and expectations associated with gender identities, which are often underrepresented in research. The challenge lies in operationalizing gender, a complex, socially constructed concept with multiple dimensions [23]. Traditional epidemiological frameworks struggle to quantify and measure gender adequately, often resulting in oversimplified representations and potentially biased findings [23]. One emerging strategy is the use of composite gender scores or indices, which employ previously-collected psychosocial variables to assess an individual’s adherence to study-specific feminine or masculine factors. However, these scores may indicate a lack of comprehensive gender considerations in study design [23]. To advance gender-sensitive research, the epidemiological community should move beyond relying solely on gender scores or solely adjusting or stratifying their analyses based on gender. Instead, complementing these indices with multiple self-reported gender measures (e.g., social support, self-efficacy, self-reported health, life stressors and lifestyle habits) representing various dimensions of gender in cohort studies would provide a more nuanced and accurate approach to gender-sensitive epidemiological research [23]. In Fig. 1 we depict a complex interplay between sex and gender factors influencing recovery and other outcomes following SCI. Whereas, depending on the research question other important sex/gender aspects may emerge. Gendered factors/relations and sex-linked biology may each be relevant independently, not relevant at all, or relevant together as synergistic factors [24]. Recent initiatives by funding agencies around the world do not mandate that scientists specifically study differences between males and females. Instead, the goal is to enhance the generalizability of studies by calculating an average effect that includes both sexes. However, if there are significant and meaningful sex differences in treatment effects, studies should be designed to ensure that these differences can be visualized and analyzed effectively. There is a possibility that as a result of these global initiatives, an increasing number of researchers will routinely perform exploratory sex subgroup analyses without considering biological or clinical relevance.

In observational studies sex (and gender) is often considered as independent variable, as an effect modifier or as a confounder, whereas, in RCTs subgroup analyses are commonly used to determine whether treatment effects vary across sexes/genders. For instance, RCTs often perform numerous subgroup analyses without correcting for multiple testing, thus, the probability of false positive claims is high [25]. Deciding whether to incorporate sex and gender considerations into study design and analyses requires careful deliberation. Conducting a systematic review of the literature before starting the original study is highly recommended [26]. This approach can help researchers identify reported sex and gender differences which can help them to justify the inclusion of sex, gender or both in their research design and data analysis, and identify relevant gender-related factors to consider in their study. Subsequently, a decision can be made if sex/gender should be considered as an independent variable, effect modifier or confounder. To assist researchers, directed acyclic graphs (DAGs) can be used for visualization of complex association between exposure and health outcome variables, as well as the confounding variables that require conditioning in the analyses [26].

In the case of observed differences, it is desirable to distinguish between clinically meaningful differences vs. clinically non-meaningful differences. The clinically meaningful differences (CMD) or minimal clinically important difference (MCID) represents the smallest benefit that holds value for persons with SCI. This concept focuses on the person’s perspective, encompassing both the extent of improvement and the significance patients attribute to that change. In case of health conditions leading to a certain level of disability, it may be of particular interest to focus on MCID in patient-reported outcomes. This is because the clinical relevance of a particular change might not be readily apparent to clinicians choosing treatments. Essentially, the MCID specifies the minimum change in an outcome that patients perceive as meaningful [27]. Often, studies lack the statistical power needed to accurately determine the existence or absence of sex/gender differences. It can also happen that association/effect estimate is significant in men but not in women, this is not evidence of sex differences, it can rather be a consequence of women's underrepresentation in a study. Even in the case of no differences between sex/gender, or null findings in one sex, presenting raw sex/gender-specific data can help avoid publication bias and facilitate future meta-analysis [28]. Thus, all authors are encouraged to include both sexes and provide sex-disaggregated results, meaning presenting the effect/association estimates per sex regardless of whether sex differences are present.

### Elucidate and interpret

Explaining the underlying pathophysiological mechanisms can foster biomarker discovery to advance high-quality clinical research in neurotherapeutics and SCI. Molecular changes mediated through sex hormones and sex-specific gene expression are a good starting point. For instance, the role of androgen hormones has been extensively studied in men with SCI, whereas the potentially protective role of female hormones such as estradiol and progesterone has been studied only in animal models of SCI. It is important to broaden the scope of research on the role of sex hormones beyond testosterone and progesterone. Dehydroepiandrosterone (DHEA) and its sulfated derivative DHEAS, the most abundant steroid hormones, have demonstrated neuroprotective effects in various experimental conditions, including models of ischemia, traumatic brain injury and SCI, but have not been studied in humans with SCI [29]. Further, a multiomics approach allows researchers to systematically study sex-biased genomic, genetic, and epigenetic landscapes in SCI. Unlike traditional hypothesis-driven studies, multiomics studies enable discovery of novel molecular changes, pathways, and targets specific to each sex [30]. Finally, the interplay between sex and gender is nicely reflected in differences in behavioral risk factor profiles related to gender (e.g. smoking or engagement in physical activity), which may have epigenetic effects that induce changes in gene expression, a mechanism usually associated with sex [31].

### Translate the evidence

Bridging basic and clinical research is key to the development of clinical guidelines and health policy changes. Translating theoretical knowledge and experimental breakthroughs into clinical practice has always been challenging [32] and it requires accumulated knowledge and reliable evidence to support decision making. Systematic reviews and meta-analyses are essential for the development of evidence-based guidelines. Recent bibliometric analysis identified major methodological limitations of published systematic reviews in the field of SCI ranging from inherent methodological flaws to low adherence to reporting guidelines [33]. The quality of underlying evidence cannot be altered, yet, for future studies the SCI research community could invest in collaborative efforts to facilitate data sharing and to provide high-quality evidence for both men and women living with SCI [33].

Specific peculiarities in SCI research limit the generalizability of current evidence and its translational potential, which merit addressing. Neurological recovery has always been the top priority topic in SCI research, yet the majority of evidence comes from pre-clinical/experimental/in vivo/in vitro studies. Specifically, sex differences in response to the injury are often studied in pre-clinical settings, and indeed, controlled experimental conditions give us the opportunity to explore biological (sex) differences in initial response to injury. Female animal models are being used in pre-clinical studies, while in clinical studies mostly men are included [9]. Women with SCI are most often middle-aged, while SCI models usually use subjects of young age or postmenopausal status. The SCI models predominantly include only transection, contusion, and compression, whereas in clinical settings injuries are more complex and often involve multiple organ injuries as a consequence of trauma [34]. This further complicates the translation of preclinical studies to clinical practice and limits their generalizability. There are misconceptions that including both sexes in research will introduce too much variability of experimental data, and requires doubling the sample size for sex-disaggregated analysis [35, 36]. We advise scientists to consult either experienced colleagues or statisticians who can help them calculate the sample size or modify the design of their experimental study to involve animals of both sexes. Studies conducted in humans remain inconclusive, as some studies show a tendency for women to experience improved recovery in measures of motor capabilities and independence [37, 38]; whereas others report higher improvement in men, or no differences between sexes in recovery after injury [39, 40]. This inconsistent evidence could be driven by differences in the type of injuries sustained in women, variations in age at the time of injury or simply by biological differences in pathophysiology of response to injuries. For instance, females tend to be injured at an older age, which can exacerbate injuries [38, 41]. On the other hand, spinal trauma at an older age is often caused by less severe mechanisms of injury (e.g., fall accidents compared to traffic or sporting accidents in younger age) which can directly influence recovery post-injury. Inconsistent evidence could also be driven by differences in methodological approaches and statistical analyses applied across studies. This again emphasizes the importance of choosing proper statistical methods to explore sex/gender differences in health outcomes. Sufficient evidence exists to suggest that the transition from limitations in functioning due to SCI to long-term disability differs between men and women [37, 38, 41]. Thus, beyond understanding biological differences in response to injury, the role of physical and social environment, through the gendered system, social position, and pathways to help, is essential to produce high-quality evidence sufficient to inform clinical or policy guidelines.

## Conclusions

Consideration of sex/gender in (SCI) research is essential to ensure that both men and women benefit from scientific advances. We developed research framework for considering and incorporating sex and gender in research using SCI as a case in point, however, the major principles in current paper can benefit everyone interested in studying sex/gender in the context of health in complex and disabling conditions, or conditions predominantly affecting one sex.

## Supplementary information

Online supplement

## Data Availability

The datasets analyzed during the current study are available from the corresponding author on reasonable request.
