# Supplementary material for: Mapping sex and gender in the landscape of spinal cord injury research: a bibliometric analysis and research framework
Source: Spinal Cord. 2025 May 29;63(7):333–41. doi: 10.1038/s41393-025-01089-7 (PMC12237693; doi:10.1038/s41393-025-01089-7)
Supplement: Supplementary file 1 — Online supplement [file 41393_2025_1089_MOESM1_ESM.docx]

**Mapping sex and gender in the landscape of spinal cord injury research: a bibliometric analysis and research framework**

Stevan Stojic^1^, Serena Affolter^2^, Gertraud Stadler^3^, Stacey A. Missmer^4^, Juergen Pannek^1,5^, Jivko Stoyanov^1,2^, Inge Eriks-Hoogland^1,5,6^, Janina Lüscher*^1,6^, Marija Glisic*^1,2^

^1^Swiss Paraplegic Research, Nottwil, Switzerland

^2^Institute of Social and Preventive Medicine (ISPM), University of Bern

^3^Gender in Medicine, Health & Human Sciences, Charité Universitätsmedizin Berlin, Berlin, Germany

^4^Department of Epidemiology, Harvard T.H. Chan School of Public Health, Harvard University, Boston, MA, USA

^5^Swiss Paraplegic Center, Nottwil, Switzerland.

^6^ Faculty of Health Sciences and Medicine, University Lucerne, Lucerne, Switzerland

*denotes equal contribution

**Correspondence:** PD Dr. M. Glisic, Swiss Paraplegic Research, Guido A. Zäch Strasse 4, 6207 Nottwil, +41 41 939 66 45, marija.glisic@paraplegie.ch

## Supplemental Table 1. Metadata quality asessment

##
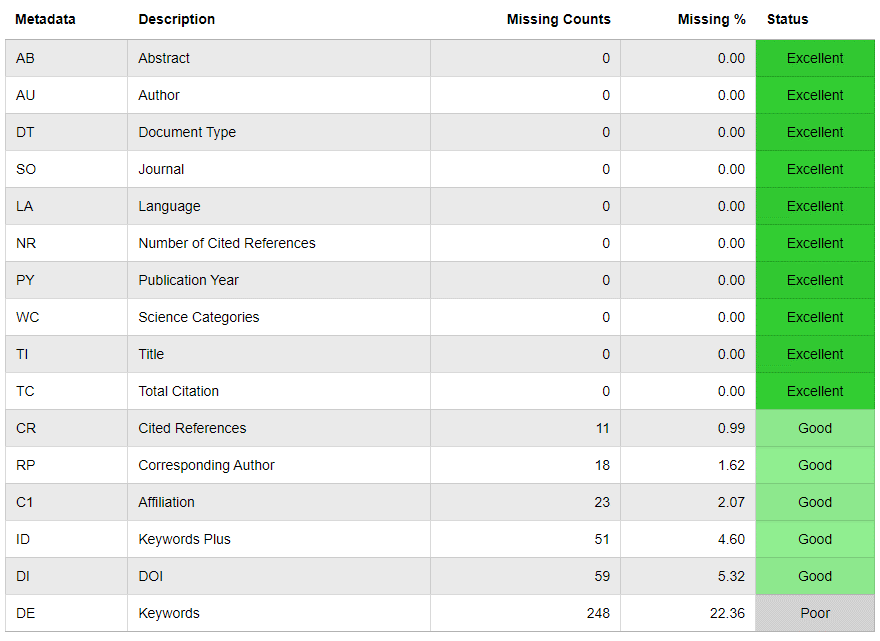


**Supplemental Table 2. Top 10 most productive countries based on corresponding author's analysis**

| **Country** | **No. of documents** | **Single country collaboration** | **Multiple country collaboration** | **Percentage of the total** |
| --- | --- | --- | --- | --- |
| **USA** | **370** | **333** | **37** | **35.9%** |
| **China** | **99** | **92** | **7** | **9.6%** |
| **Canada** | **75** | **49** | **26** | **7.3%** |
| **Japan** | **44** | **43** | **1** | **4.3%** |
| **Switzerland** | **39** | **21** | **18** | **3.8%** |
| **Netherlands** | **33** | **27** | **6** | **3.2%** |
| **Germany** | **27** | **18** | **9** | **2.6%** |
| **Australia** | **26** | **19** | **7** | **2.5%** |
| **Iran** | **25** | **18** | **7** | **2.4%** |
| **Sweden** | **25** | **19** | **6** | **2.4%** |

| **Supplemental Table 3. Top 10 most cited documents** | | | |
| --- | --- | --- | --- |
| **Document title, authors and journal** | **Total Citations** | **Total citations per Year** | **Normalized total citations^1^** |
| GBD 2016 Neurology Collaborators. Global, regional, and national burden of neurological disorders, 1990-2016: a systematic analysis for the Global Burden of Disease Study 2016. Lancet Neurol. 2019 May;18(5):459-480. doi: 10.1016/S1474-4422(18)30499-X. Epub 2019 Mar 14. PMID: 30879893; PMCID: PMC6459001. | **1672** | **334.40** | **28.32** |
| GBD 2016 Traumatic Brain Injury and Spinal Cord Injury Collaborators. Global, regional, and national burden of traumatic brain injury and spinal cord injury, 1990-2016: a systematic analysis for the Global Burden of Disease Study 2016. Lancet Neurol. 2019 Jan;18(1):56-87. doi: 10.1016/S1474-4422(18)30415-0. Epub 2018 Nov 26. Erratum in: Lancet Neurol. 2021 Dec;20(12):e7. PMID: 30497965; PMCID: PMC6291456. | **1672** | **334.40** | **28.32** |
| Wyndaele M, Wyndaele JJ. Incidence, prevalence and epidemiology of spinal cord injury: what learns a worldwide literature survey? Spinal Cord. 2006 Sep;44(9):523-9. doi: 10.1038/sj.sc.3101893. Epub 2006 Jan 3. PMID: 16389270. | **729** | **40.50** | **8.77** |
| Nagai M, Usuku K, Matsumoto W, Kodama D, Takenouchi N, Moritoyo T, Hashiguchi S, Ichinose M, Bangham CR, Izumo S, Osame M. Analysis of HTLV-I proviral load in 202 HAM/TSP patients and 243 asymptomatic HTLV-I carriers: high proviral load strongly predisposes to HAM/TSP. J Neurovirol. 1998 Dec;4(6):586-93. doi: 10.3109/13550289809114225. PMID: 10065900. | **496** | **19.08** | **4.55** |
| Jain NB, Ayers GD, Peterson EN, Harris MB, Morse L, O'Connor KC, Garshick E. Traumatic spinal cord injury in the United States, 1993-2012. JAMA. 2015 Jun 9;313(22):2236-43. doi: 10.1001/jama.2015.6250. PMID: 26057284; PMCID: PMC4712685. | **391** | **43.44** | **11.52** |
| Mitchell GS, Johnson SM. Neuroplasticity in respiratory motor control. J Appl Physiol (1985). 2003 Jan;94(1):358-74. doi: 10.1152/japplphysiol.00523.2002. PMID: 12486024. | **310** | **14.76** | **4.66** |
| Jackson AB, Dijkers M, Devivo MJ, Poczatek RB. A demographic profile of new traumatic spinal cord injuries: change and stability over 30 years. Arch Phys Med Rehabil. 2004 Nov;85(11):1740-8. doi: 10.1016/j.apmr.2004.04.035. PMID: 15520968. | **298** | **14.90** | **4.22** |
| Fouts DE, Pieper R, Szpakowski S, Pohl H, Knoblach S, Suh MJ, Huang ST, Ljungberg I, Sprague BM, Lucas SK, Torralba M, Nelson KE, Groah SL. Integrated next-generation sequencing of 16S rDNA and metaproteomics differentiate the healthy urine microbiome from asymptomatic bacteriuria in neuropathic bladder associated with spinal cord injury. J Transl Med. 2012 Aug 28;10:174. doi: 10.1186/1479-5876-10-174. PMID: 22929533; PMCID: PMC3511201. | **293** | **24.42** | **7.79** |
| Hu R, Mustard CA, Burns C. Epidemiology of incident spinal fracture in a complete population. Spine (Phila Pa 1976). 1996 Feb 15;21(4):492-9. doi: 10.1097/00007632-199602150-00016. PMID: 8658254. | **284** | **10.14** | **3.48** |
| Olby N, Levine J, Harris T, Muñana K, Skeen T, Sharp N. Long-term functional outcome of dogs with severe injuries of the thoracolumbar spinal cord: 87 cases (1996-2001). J Am Vet Med Assoc. 2003 Mar 15;222(6):762-9. doi: 10.2460/javma.2003.222.762. PMID: 12675299. | **215** | **10.24** | **3.23** |
| **^1^Calculated by dividing the actual count of citing items by the expected citation rate for documents with the same year of publication.** | | | |

**Supplemental Figure 1. Annual scientific production**

**
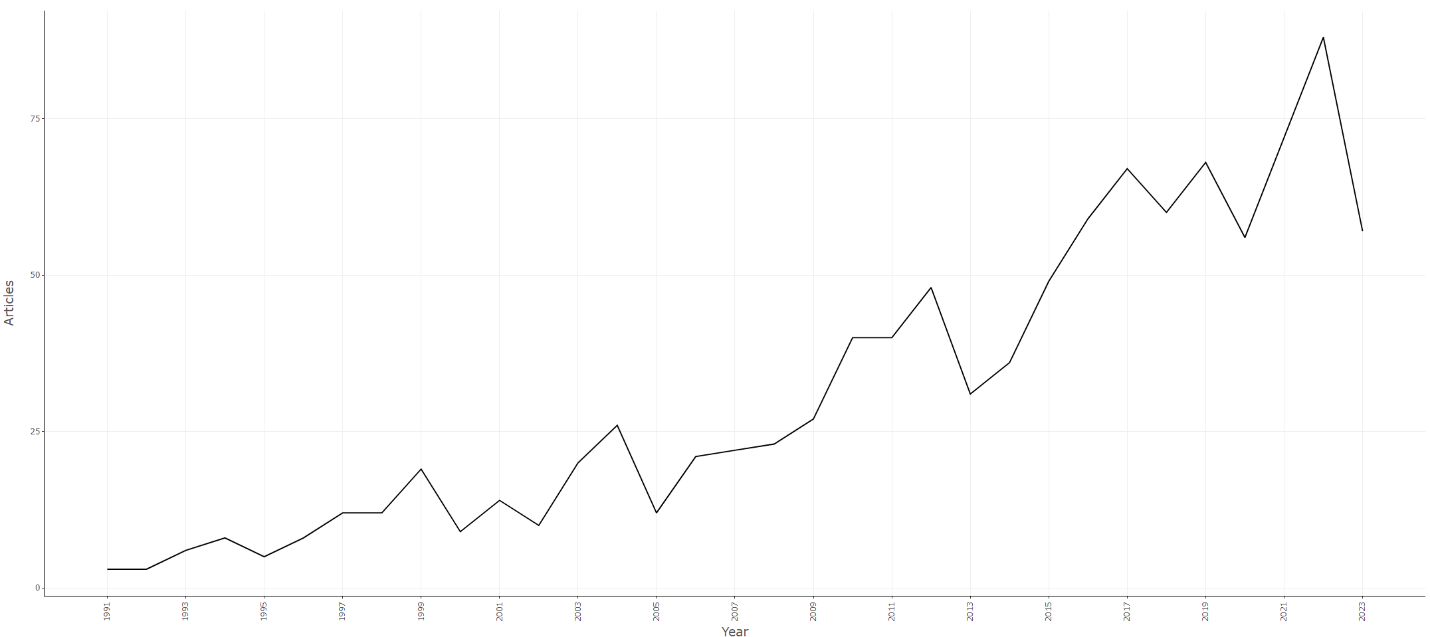
**

***Annual Growth Rate was 9.64%**

**Supplemental Figure 2. Key sources, authors and institutions**

**a) Top 10 most productive sources**

**
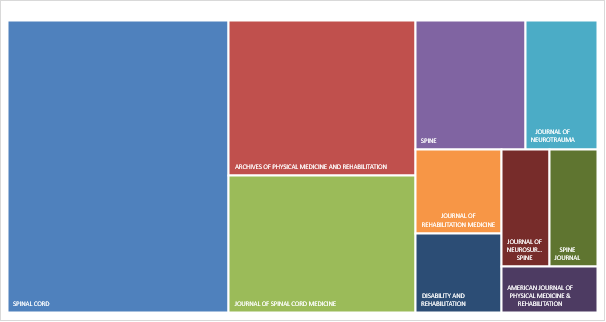
**

**b) Top 10 most productive institutions**

**
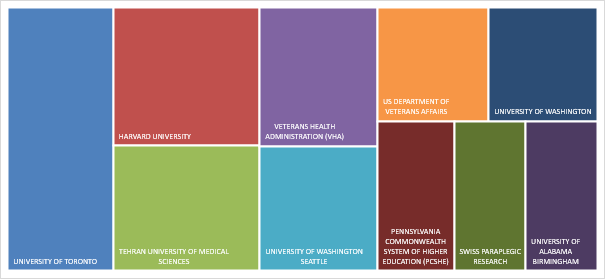
**

**c) Top 10 most productive authors**

**
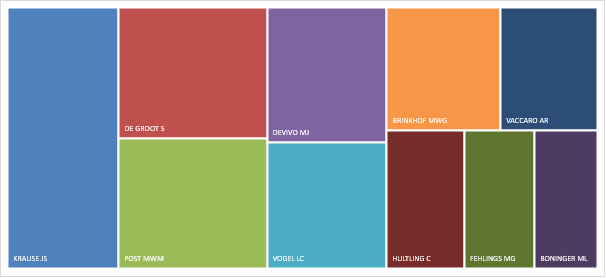
**

**Supplemental Figure 3. Analysis of research themes**

## a) Top 50 authors' keywords

**
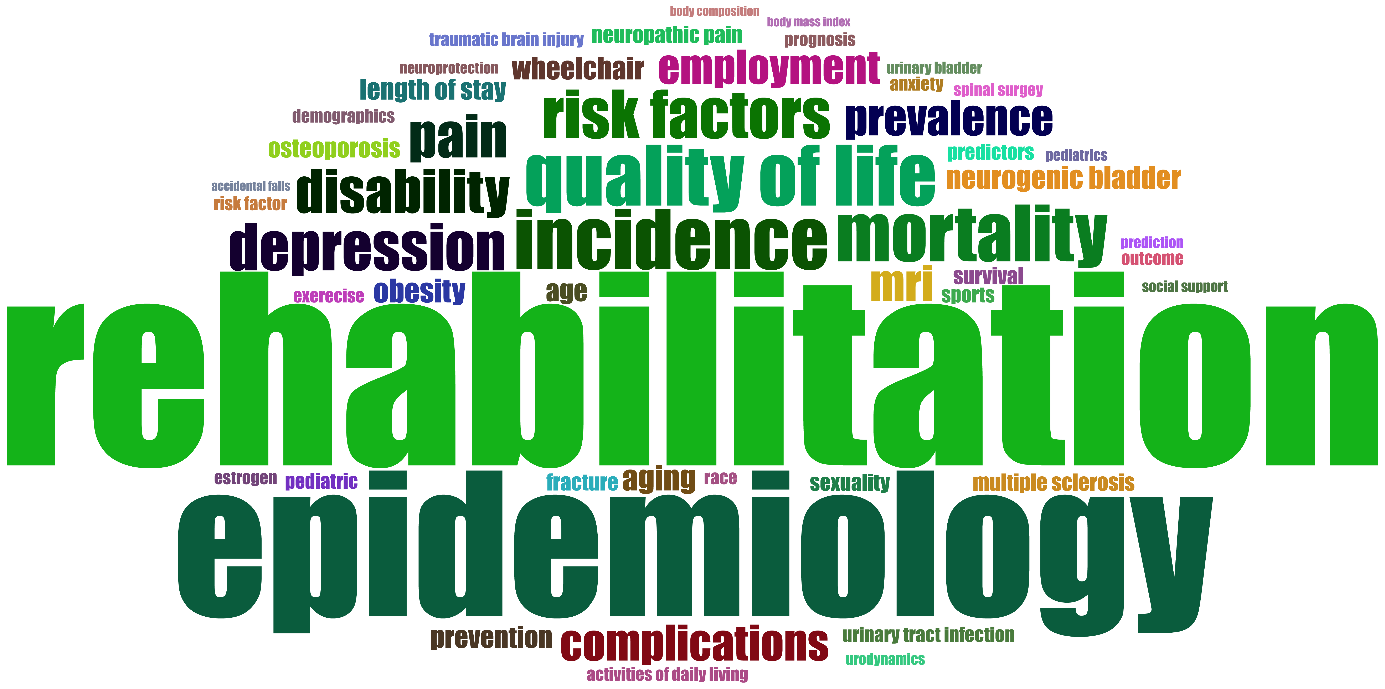
**

**b) Thematic map of author's keywords**

**
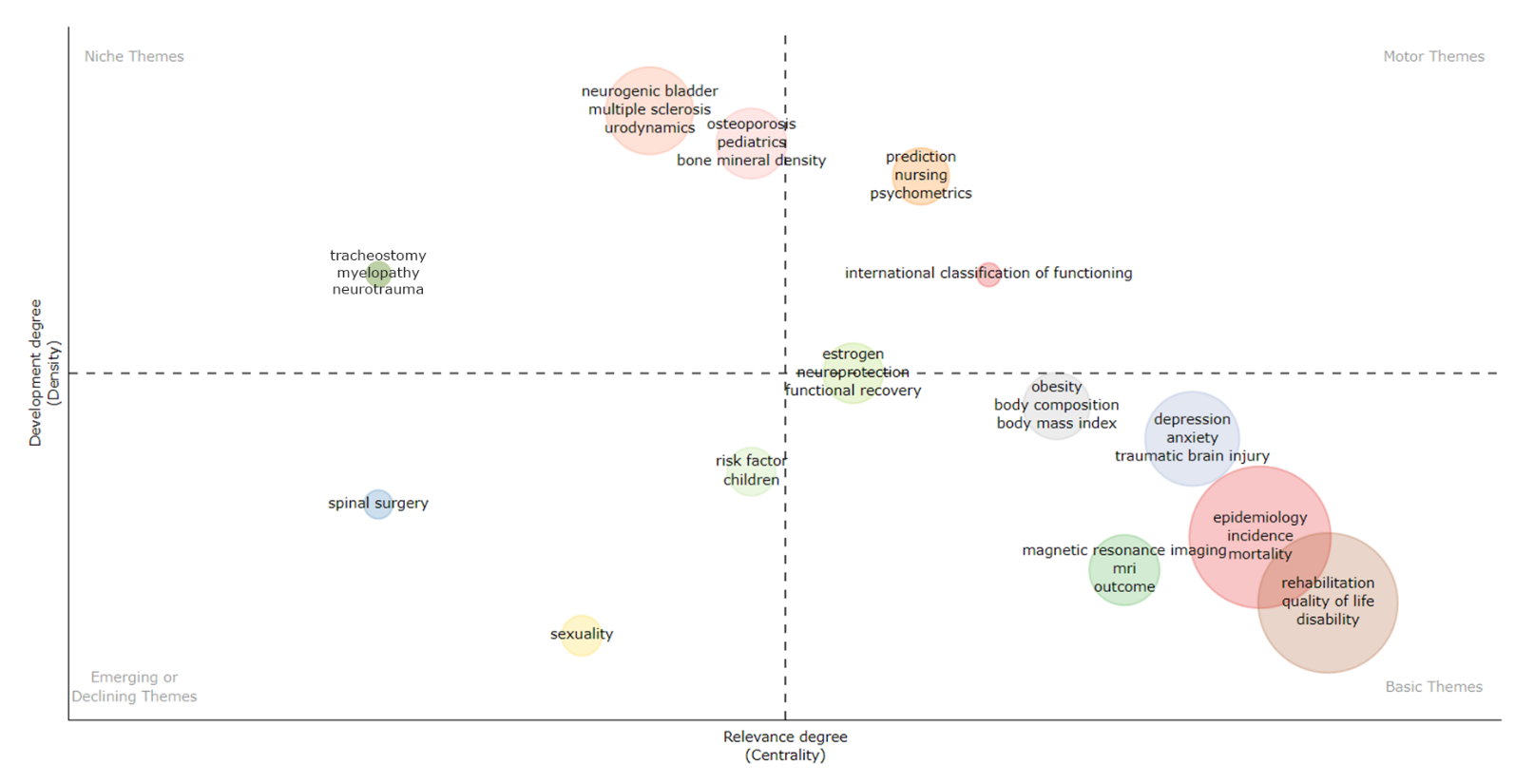
**

*The size of the cluster is given by the number of occurrences of the keywords that it contains and therefore by the number of linked papers. The label chosen by the software corresponds to the predominant keyword*

**Appendix I.** Search strategy

Database: Web of Science

Date of search: 05.10.2023

"TS=(((spine OR spinal OR vertebr*) NEAR/3 (trauma* OR injur* OR damag* OR wound*))

OR ("spinal cord" NEAR/3 (disease* OR contusi* OR laceration* OR transection* OR

lesion* OR trauma* OR ischemi* OR ischaemi*)) OR (myelopath* NEAR/3 (trauma* OR

post-trauma* OR posttrauma*)) OR (SCI-group*) OR "central cord injury syndrome*" OR

"central cord syndrome*" OR "central spinal cord syndrome*" OR "cauda equine

syndrome*" OR "anterior cord syndrome*" OR "conus medullaris syndrome*" OR "Brown

Sequard*" OR paraplegi* OR quadriplegi* OR tetraplegi*)

AND

TS=( gender* OR sexes OR ((sex*) NEAR/3 (differ* OR dimorphism* OR dichromatism* OR

role* OR specific* OR depend* OR characteristic*)) OR (biologic* NEAR/3 sex*))

Number of hits: n= 3,674

Restricted to reviews and articles, Number of hits: n=3,611

Search query: https://www.webofscience.com/wos/woscc/summary/bc80aa99-faec-4060-

bd7d-c2a59246f206-a85cec60/relevance/1
